# Investigation of GeSn Strain Relaxation and Spontaneous Composition Gradient for Low-Defect and High-Sn Alloy Growth

**DOI:** 10.1038/s41598-018-24018-6

**Published:** 2018-04-04

**Authors:** Wei Dou, Mourad Benamara, Aboozar Mosleh, Joe Margetis, Perry Grant, Yiyin Zhou, Sattar Al-Kabi, Wei Du, John Tolle, Baohua Li, Mansour Mortazavi, Shui-Qing Yu

**Affiliations:** 1 Department of Electrical Engineering, University of Arkansas, Fayetteville, AR 72701 USA; 2 Institute for Nanoscience and Engineering, University of Arkansas, Fayetteville, AR 72701 USA; 3 Microelectronics-Photonics Program, University of Arkansas, Fayetteville, AR 72701 USA; 4 Department of Electrical Engineering, Arkansas Tech University, Russellville, AR 72801 USA; 5 ASM, 3440 East University Drive, Phoenix, AZ 85034 USA; 6 Arktonics, LLC, 1339 South Pinnacle Drive, Fayetteville, AR 72701 USA; 7 Department of Chemistry & Physics, University of Arkansas at Pine Bluff, Pine Bluff, AR 71601 USA; 8 Department of Electrical Engineering, Wilkes University, 84 West South Street, Wilkes-Barre, PA 18766 USA

## Abstract

Recent development of group-IV alloy GeSn indicates its bright future for the application of mid-infrared Si photonics. Relaxed GeSn with high material quality and high Sn composition is highly desirable to cover mid-infrared wavelength. However, its crystal growth remains a great challenge. In this work, a systematic study of GeSn strain relaxation mechanism and its effects on Sn incorporation during the material growth via chemical vapor deposition was conducted. It was discovered that Sn incorporation into Ge lattice sites is limited by high compressive strain rather than historically acknowledged chemical reaction dynamics, which was also confirmed by Gibbs free energy calculation. In-depth material characterizations revealed that: (i) the generation of dislocations at Ge/GeSn interface eases the compressive strain, which offers a favorably increased Sn incorporation; (ii) the formation of dislocation loop near Ge/GeSn interface effectively localizes defects, leading to the subsequent low-defect grown GeSn. Following the discovered growth mechanism, a world-record Sn content of 22.3% was achieved. The experiment result shows that even higher Sn content could be obtained if further continuous growth with the same recipe is conducted. This report offers an essential guidance for the growth of high quality high Sn composition GeSn for future GeSn based optoelectronics.

## Introduction

Group-IV alloy GeSn has drawn great attentions as a complementary metal–oxide–semiconductor (CMOS) compatible optoelectronic material for Si photonics^1–3^. The devices based on GeSn alloy could be monolithically integrated into well-established and high-yield Si integrated circuits, which is favorable for chip-scale Si photonics featuring smaller size, lower cost, and higher reliability. GeSn with Sn composition over 8% could become direct bandgap material^4–7^, which is advantageous for efficient light source. Moreover, higher Sn incorporation and complete strain relaxation are essential to increase the bandgap directness and extend the wavelength coverage well into the mid infrared range.

From material growth perspective, the epitaxy of relaxed high Sn composition GeSn with high material quality is challenging due to the following factors: (i) Low (<1%) equilibrium solid solubility of α-Sn in Ge^8^: Sn atoms tend to segregate during the epitaxy growth and form β-Sn clusters^9,10^; (ii) Small temperature window for GeSn epitaxy growth: Low temperature is required for high Sn incorporation to suppress Sn precipitation. However, low temperature growth easily leads to epitaxial breakdown for thick film due to the severe surface roughening^11^; (iii) Large lattice mismatch (~15%) between Ge and α-Sn, making relaxed GeSn epitaxy on Ge difficult. Large amount of misfit dislocations (MDs) will be introduced into GeSn, including edge dislocations, threading dislocations, stacking faults, and twin boundaries. Particularly, threading dislocations, unless formed as loops, would glide and propagate through the entire epitaxial layer. Threading segments have very limited contribution for strain relaxation but could severely deteriorate material quality by acting as non-radiative recombination centers.

Benefiting from the maturity of epitaxial technology, such as molecular beam epitaxy (MBE), and chemical vapor deposition (CVD), high quality single crystal GeSn could be grown under non-equilibrium conditions. MBE has achieved single crystalline GeSn on Si^12^, Ge^11,13,14^, and other substrates, leading to the demonstration of light emitting diodes (LEDs)^15^ and photo detectors^16^. However, the epitaxy of high Sn content GeSn with decent quality to show clear photoluminescence (PL) spectra is still under development. Recently, CVD growth of GeSn has made significant progress by using industry standard manufacture technique^17–23^. High quality GeSn with 12.6% Sn content has been grown using Ge_2_H_6_ and SnCl_4_ as precursors^17^, which has enabled the first demonstration of optical pumping GeSn laser with an operating temperature up to 90 K^18^. The lasing of GeSn micro-disks with 16% Sn has also been realized with a wavelength up to 3.1 μm at 180 K by using the same growth chemistry^19^. High order Ge hydrides such as Ge_3_H_8_ or Ge_4_H_10_ were also utilized for GeSn growth in order to pursue high Sn incorporation^20^. Those highly reactive Ge hydrides enable the high growth rate at low temperature due to their weak Ge-Ge molecular bond to favor more Sn incorporation. However, utilizing GeH_4_ as precursor for GeSn growth remains very attractive for industrial manufacturing due to its low cost and high thermal stability at room temperature^21–24^. We previously reported low-defect and thick GeSn growth with Sn incorporation up to 17% using GeH_4_ and SnCl_4_ as precursors^21,22^. The optically pumped GeSn edge-emitting lasers using these GeSn materials demonstrated a broad wavelength coverage of 2–3 μm and the highest lasing temperature of 180 K^23^.

Historically it is generally acknowledged that the Sn incorporation via CVD epitaxy growth of GeSn is limited by chemical reaction dynamics. Therefore, substantial growth efforts were devoted to the process optimization of surface chemistry kinetics and thermodynamics^20,24,25^. However, recently we discovered a spontaneous-relaxation-enhanced (SRE) Sn incorporation mechanism for growth using GeH_4_ and SnCl_4_ as precursors^23^. It was found that Sn incorporation is primarily limited by compressive strain under Sn oversaturation condition while surface chemical reaction being secondary^26^. Since Sn exhibits lower free energy^10^, the excess provided Sn atoms will float and segregate on the surface, or be desorbed from the surface. For example, when the nominal growth recipe was used with targeting Sn content of 12%, the Sn incorporation starts from 12% and then increases continuously to 15% due to the material gradual relaxation. More Sn incorporation also results in the reduction of surface Sn segregation. Since all the growth parameters maintained invariable, the gradient GeSn was grown spontaneously rather than intentionally. Guided by SRE discovery, new growth strategies were carefully designed which lead to high quality and high Sn incorporation^23^. Other research group also observed SRE mechanism in the study of GeSn epitaxy^27^. While this approach showed its effectiveness based on the previous work, the microscopic mechanism of GeSn strain relaxation induced high Sn incorporation as well as high quality material formation is still unclear. A thorough understanding of the mechanism would provide great insights to guide the future high quality and high Sn composition GeSn material growth for the development of high performance Si based optoelectronics.

In this work, a systematic study of strain relaxation mechanism for CVD grown GeSn and its effects on Sn incorporation during the GeSn growth process was performed. This study fills the blanks of in-depth understanding of SRE mechanism. It is revealed that the generation of dislocations at GeSn/Ge interface accommodates the large lattice mismatch and favors the crystalline nucleation for initial GeSn growth. A self-organized dislocation network is formed within the first 200–300 nm GeSn layer near the GeSn/Ge interface, which blocks the propagation of dislocation, leaving the subsequent GeSn layer low defect. In addition, the spontaneous gradient Sn incorporation was generated at entire GeSn layer due to the compressive strain relaxation. This paper is organized as the following: First of all, the sample growth methodology for high Sn incorporation was presented and the detailed growth results were summarized. One sample has reached a world-record final Sn composition of 22.3%, which significantly breaks the previously reported Sn incorporation limit even for using high order Ge hydrides as precursors; Second, following the growth sequence, the dislocation configuration at GeSn/Ge interface was investigated using transmission electron microscopy (TEM) to study the initial dislocation generation; Third, the formation of dislocation network region beyond the initial critical thickness was examined to understand the mechanism of dislocation network efficiently localizing dislocations and preventing the upward propagation of threading dislocations; Finally, the Gibbs free energy model was used to further discuss the relationship between compressive strain and Sn incorporation; Based on the mechanism discovered in this work, the approach to achieve higher Sn composition was proposed.

## Results

### Growth methodology and sample characteristics

GeSn samples were grown on relaxed Ge buffered Si substrate, using ASM Epsilon^®^ 2000 Plus reduced pressure CVD (RPCVD) system with GeH_4_ and SnCl_4_ precursors (see methods). Two strategies have been used in GeSn epitaxy to obtain high Sn incorporation: (i) the SRE approach for sample A, B and C with nominal growth recipe of 9%, 10% and 11% Sn and the corresponding finally achieved Sn compositions 12.5%, 12.9% and 15.9%, respectively; (ii) The GeSn virtual substrate (VS) approach for sample D and E in which GeSn VS was prepared through SRE approach with a nominal growth recipe of 12% and achieved intermediate Sn composition of 16.5% for both samples. Sample D was then grown with single-step Sn enhanced recipe on GeSn VS with the final achieved Sn composition 17.5%. Sample E was grown with a three-step gradient GeSn recipe (see methods). For each step the grading rate of Sn incorporation was designed to be moderate in order to suppress the breakdown of continuous growth. The graded structure eases compressive strain gradually, leading to the continuous increase of Sn concentration. The final Sn content was obtained as 22.3%, an unprecedented achievement so far for CVD technology.

**Figure 1:**
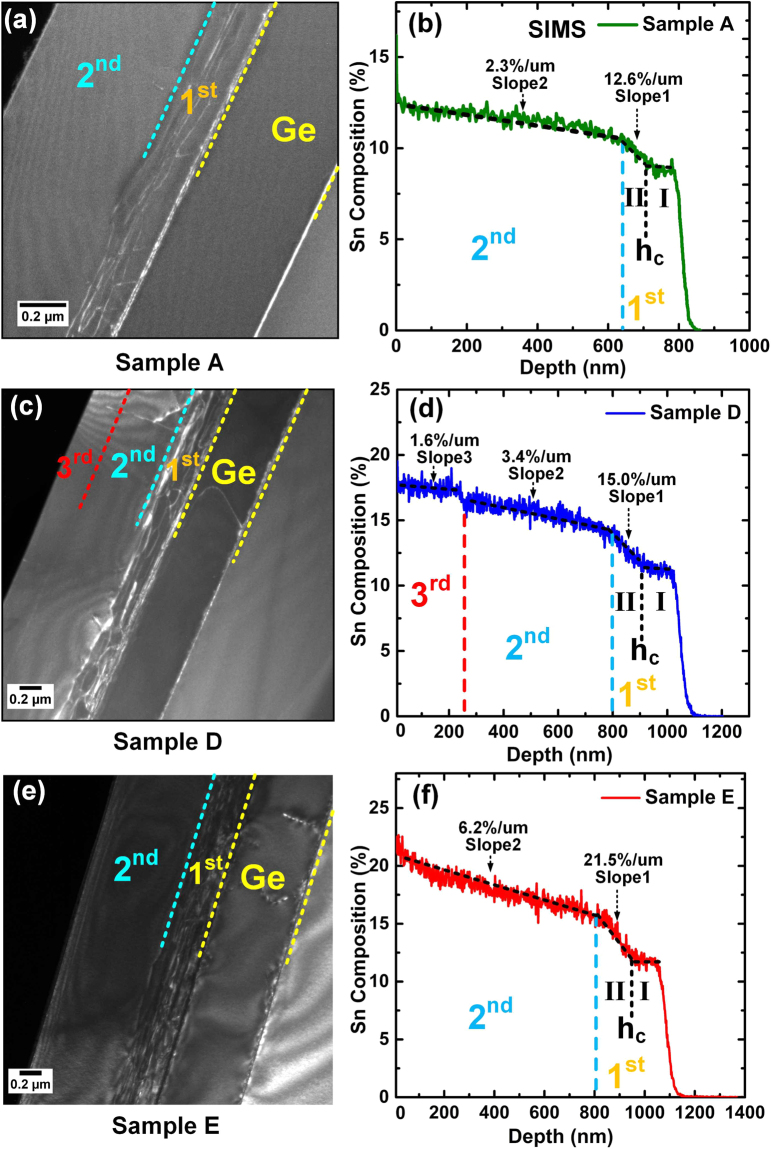
(**a**) Dark field TEM image of sample A shows the defective GeSn (1^st^ layer) and low defect GeSn (2^nd^ layer), respectively. (**b**) SIMS of sample A indicates the spontaneous Sn gradient GeSn at both layers with different gradient rates. The 1^st^ layer was subdivided into region-I and II with boundary of critical thickness *h*_*c*_. (**c**) In TEM Image of sample D, an additional GeSn (3^rd^ layer) is shown on top of two-layer structure. One bright line travels across the 2^nd^ and 3^rd^ layer indicates the penetration of threading dislocation. (**d**) SIMS of sample D shows the 3^rd^ layer with up to 17. 5% Sn content. (**e**) For TEM image of sample E, the boundaries between each Sn-enhanced step are indistinguishable in the 2^nd^ layer. (**f**) In SIMS of sample E, Sn incorporation increases continuously in the 2^nd^ layer. The Maximum Sn content of 22. 3% was achieved.

The material characterizations were performed after the growth. Table 1 summarizes layer thicknesses, Sn compositions, compressive strains and degrees of relaxation for five samples. Typical dark field TEM images and Secondary Ion Mass Spectrometry (SIMS) of sample A, D, E are presented in Fig. 1(a,c and e) and (b,d and f), respectively. Samples B and C show similar results as sample A (Supplementary section 1). SIMS results of Sn composition were cross checked by Energy-dispersive X-ray Spectroscopy (EDX) with good agreement (Supplementary section 1). Strains and degree of relaxation were studied by data fitting of reciprocal space mapping (RSM) of X-ray diffraction (XRD) (Supplementary section 2).

**Table 1:** Summary of GeSn layer thicknesses, the maximum Sn compositions, average of Sn compositions, degree of compressive strains and degree of relaxations.

Sample	Structure	Thickness (nm)	The maximum Sn Composition (%) Region I/II	Average Sn Composition by XRD (%)	Strain/relaxation (%)
A	1^st^ layer	180	8.8/10.2	9.4	−0. 04/96.5
	2^nd^ layer	660	12.5	11.4	−0. 14/63.9
B	1^st^ layer	320	9. 2/10.5	9.7	−0. 24/80.2
	2^nd^ layer	500	12.9	12.1	−0. 39/30.7
C	1^st^ layer	250	11.7/13.2	10.5	−0. 01/92.4
	2^nd^ layer	670	15.9	14.4	−0. 29/52.1
D	1^st^ layer	310	11. 2/13.7	11.9	−0. 04/94.7
	2^nd^ layer	550	16. 5	15.5	−0. 28/60.0
	3^rd^ layer	260	17. 5	17.4	−0. 38/3.8
E	1^st^ layer	380	11.9/15.5	12.3	−0. 01/93.7
	2^nd^ layer	830	22. 3	19.0	−0. 61/2.8

For sample A, two GeSn layers could be clearly resolved as shown in TEM images of Fig. 1(a): (i) highly defective layer (1^st^ layer) of 180 nm thickness on Ge buffer and (ii) low defect layer (2^nd^ layer) of 660 nm thickness above the 1^st^ layer. Correspondingly, the two-layer structure observed from TEM image was also marked on the SIMS plot. From SIMS, both layers show Sn composition spontaneously enhanced gradient. The 1^st^ layer could be further subdivided into two regions defined by the boundary of the critical thickness *h*_*c*_, in which region-I and II represent constant low Sn content and Sn-enhanced gradient, respectively. Hereby the critical thickness *h*_*c*_ was calculated based on People – Bean (P-B) model^28^ (Supplementary section 3). The region I maintains with 8.8% Sn contents while the region II obtains the maximum 10.2% Sn contents with sharp Sn composition gradient. Through linear data fitting the gradient rate at region II of the 1^st^ layer was obtained as 12.6%/μm, which is 5.5 times of 2.3%/μm in the 2^nd^ layer. Note that since the 2^nd^ layer was Sn gradient with a small gradient rate, part of the strain could be relaxed by the biaxial distortion of lattice constant without breaking the cubic crystal structure.

For Sample D, the GeSn VS exhibits a similar two-layer structure with the thickness of 310 nm and 550 nm for the 1^st^ and 2^nd^ layer, respectively. The additional 3^rd^ layer was measured with a thickness of 260 nm, which is below the theoretical critical thickness of 1171 nm calculated by P-B model (Supplementary section 3). Therefore, the 3^rd^ layer of sample D could be considered as pseudomorphic growth which was further confirmed by RSM of XRD analysis (Supplementary section 2). One bright line was observed to travel across the 2^nd^ and 3^rd^ layers, indicating the penetration of threading dislocation which is partly responsible for strain relaxation. From SIMS, the two-layer structure was marked and the 1^st^ layer was divided into region-I and II, similar to sample A. Region I shows the constant Sn composition as 11.2% while region II reaches the final Sn composition of 13.7%. The gradient rate at region II of the 1^st^ layer and the 2^nd^ layer are 15%/μm and 3.4%/μm, respectively, which is higher than that of sample A. At the 3^rd^ layer the Sn composition was slightly enhanced with gradient rate of 1.6%/μm. Eventually 17.5% Sn incorporation was achieved on top of the 3^rd^ layer.

For sample E, the GeSn VS shows a similar two-layer structure with sample D. The 1^st^ defective layer was observed with thickness of 380 nm and the 2^nd^ layer of 830 nm thickness is low defect without distinct boundaries between the steps, suggesting the smooth growth transition. From SIMS, the two-layer structure and region-I and II in the 1^st^ layer were marked. The Sn content at region I is 11.9% and the final Sn content at region II is 15.5%. The gradient rate at region II and the 2^nd^ layer is 21.5%/μm and 6.2%/μm, both of which are higher than sample A and sample D. Sample E achieved the final world-record Sn composition of 22.3% after three-step gradient growth. It is noteworthy that based on the sharp gradient rate at the end of 2^nd^ layer, higher Sn incorporation than the value of 22.3% is expected if sample E could be grown thicker with the same recipe. High Sn content of sample E increases light emission efficiency due to more directness of bandgap and extends operating wavelength up to 3220 nm, evidenced by photoluminescence spectra shown in Supplementary section 4.

### Dislocation configuration at GeSn/Ge interface

**Figure 2:**
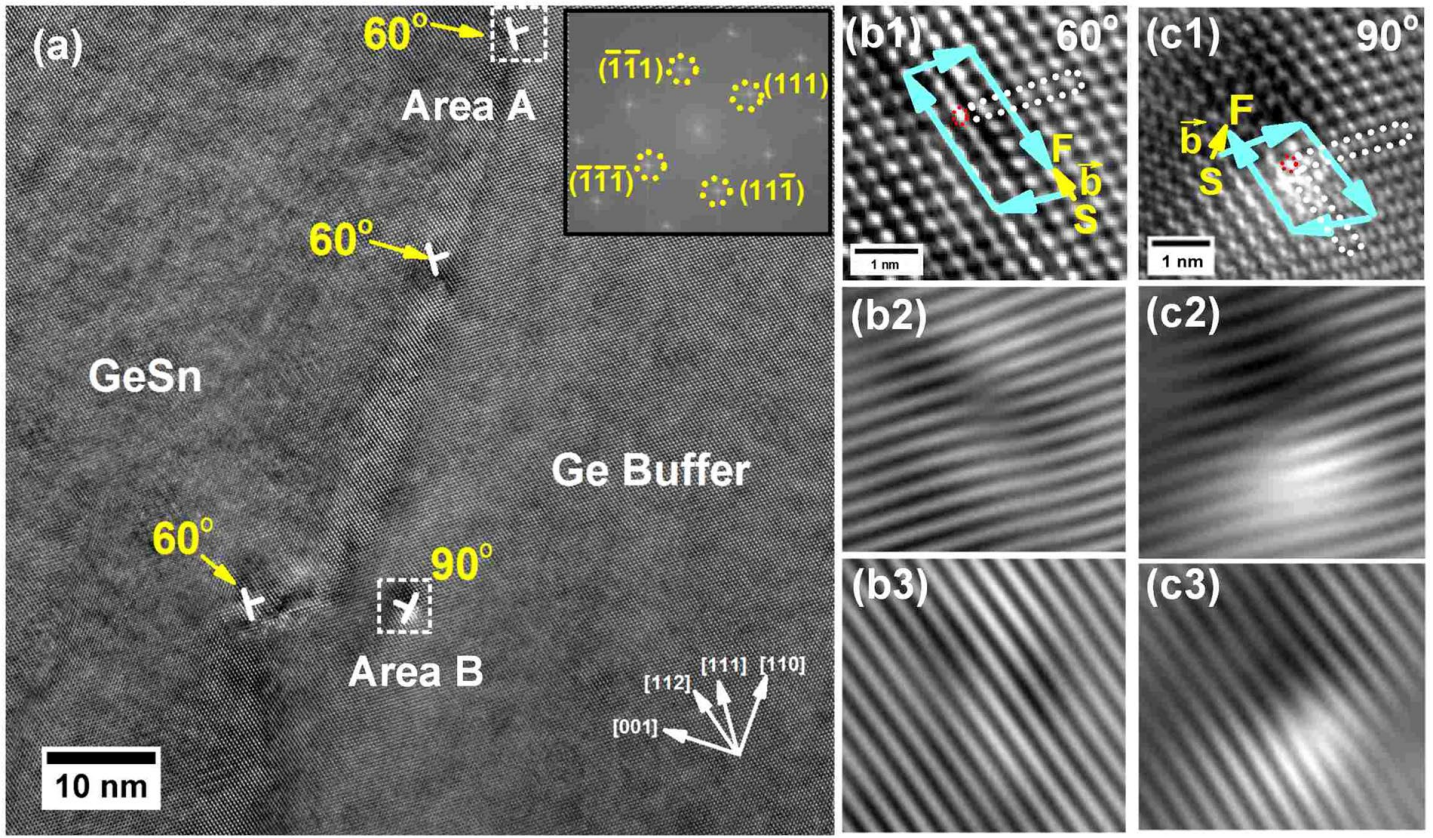
(**a**) The HRTEM image at the GeSn/Ge interface of sample B viewed from $[\bar{1}10]$ direction. The mixed type 60° dislocations dominate at the interface due to the low activation energy. The inset shows FFT pattern with different planes marked. (**b1**) Zoomed-in image of area A shows atomic core structure of 60° dislocation. Burger circuit of 60° dislocation was drawn, identifying its projected burger vector is $\frac{a\,}{4}[112]$ in $(\bar{1}10)\,$plane. The inverse FFT images were obtained by masking (**b2**) $\{(111)(\bar{1}\bar{1}\bar{1})\}\,$and (**b3**) $\{(11\bar{1})(\bar{1}\bar{1}1)\}$ planes in FFT, showing the extra (111) plane. (**c1**) Zoomed-in image of area B indicates core structure of Lomer dislocation. Burger circuit was drawn to identify burger vector$\,\,\frac{a\,}{2}[110]\,$in (001) plane. Inverse FFT images of Lomer dislocation by masking (**c2**) $\{(111)(\bar{1}\bar{1}\bar{1})\}\,$and (**c3**) $\{(11\bar{1})(\bar{1}\bar{1}1)\}$ planes in FFT were also shown.

**Figure 3:**
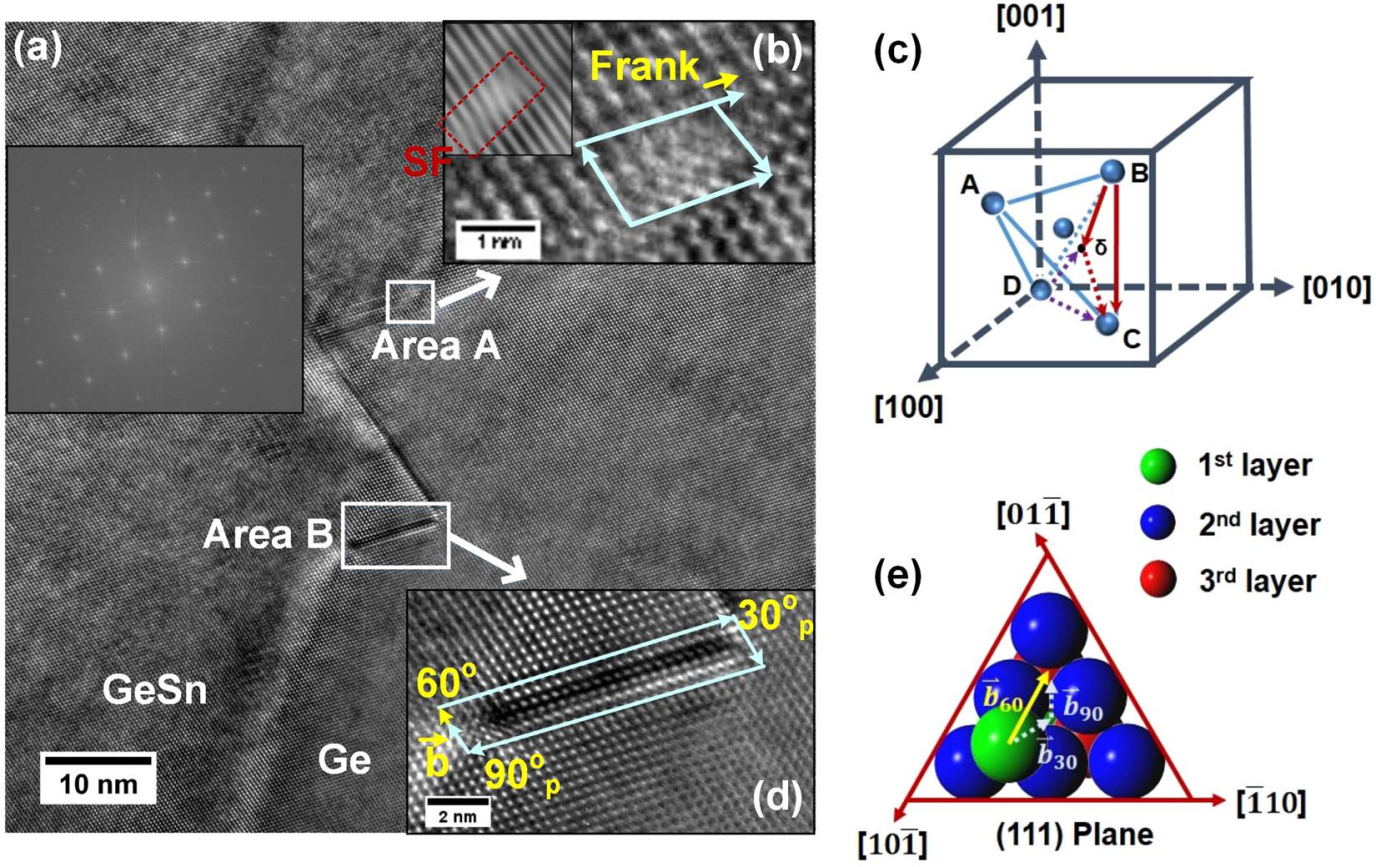
(**a**) Zigzagged intrinsic stacking faults at the GeSn/Ge interface of sample B, correspond to two dislocation reactions. The inset shows FFT pattern in which the presence of Streaks along [111] direction indicates formation of stacking faults. (**b**) Zoomed-in TEM image of area A presents Frank partial dislocation associated with stacking fault (SF). The insert is inverse FFT images by masking $\{(11\bar{1})(\bar{1}\bar{1}1)\}$ planes in FFT pattern. The stacking fault is terminated by the reaction of Frank and Shockley partial dislocation. Lomer dislocation is formed after the reaction. (**c**) Thompsons tetrahedron diagram was drawn, on which two mechanisms of stacking faults are marked: i) $D\delta +\delta C\to DC$ and ii)$\,BC\to B\delta +\delta C$. (**d**) In zoomed-in image of area B, the 60° dislocation dissociates into 30° and 90° Shockley partial dislocation pairs, bound by stacking fault. (**e**) The glide motion of top atoms in (111) plane was illustrated, corresponding to dissociation of 60° dislocation: ${\vec{b}}_{60}^\circ \to {\vec{b}}_{30}^\circ +{\vec{b}}_{90}^\circ$.

The current study of dislocation configuration at GeSn/Ge interface is preliminary while the relaxation process at the interface for other group IV epitaxy such as Ge or SiGe on Si has been extensively studied. In this section, the GeSn/Ge interface was investigated by using the high resolution TEM (HRTEM) technique to probe the dislocation configuration, which could microscopically reveal the accommodation mechanism of large lattice mismatch at the interface for the initial GeSn nucleation. Through the analysis of atomic level TEM at the interface, it was found that perfect 90° pure edge (Lomer) and 60° mixed dislocations are formed and the 60° mixed dislocations are dominant over Lomer dislocations at the interface, as shown in Fig. 2. Intrinsic stacking faults were also observed, which are associated with two different reactions of dislocations: 60° dislocation dissociation and Lomer dislocation formation, as shown in Fig. 3. By introducing dislocations, the compressive strain near the interface is partially relaxed, favoring the initial crystalline nucleation of GeSn on Ge buffer.

The dislocation generation at the GeSn/Ge interface was studied for each sample with sample B as a typical example presented in this paper. Both Lomer and 60° mixed dislocations are formed near the interface, as shown in bright field HRTEM image viewed from $[\bar{1}10]$ direction in Fig. 2(a). The observation of existence of Lomer dislocation was highly consistent with other studies, even though different CVD reactor and precursors were utilized. From the image, it is clear that the 60° dislocations outnumbered the Lomer dislocations, which indicates that the 60° dislocations are dominant for the low-temperature (<400 °C) GeSn epitaxy on Ge. This is because the activation energy of 60° dislocations is lower than that of Lomer dislocations since the gliding will proceed through the switching inter-atomic bonds instead of the diffusion motion of individual atoms^29^. The inset shows fast Fourier transform (FFT) pattern with different planes marked. In order to verify 60° dislocation, magnified high resolution TEM images of area A is shown in Fig. 2(b1), exhibiting the typical core structure of 60° dislocation. Its burger vector is identified as $\frac{a\,}{4}[112]$ by drawing the burger circuit encircling the dislocation core. It is equal to the projected component of burger vector $\frac{a\,}{2}[10\bar{1}]$ of 60° dislocation in the projection of $(\bar{1}10)$ plane. The corresponding inverse FFT images are obtained by masking $\{(111)(\bar{1}\bar{1}\bar{1})\}$and $\{(11\bar{1})(\bar{1}\bar{1}1)\}$ planes in FFT patterns, as shown in Fig. 2(b2) and (b3), respectively. The extra half plane of 60° dislocation was observed to be located at (111) plane. The magnified high resolution TEM image of area B presents core structure of Lomer edge dislocation, as shown in Fig. 2(c1). The corresponding burger vector of $\frac{a\,}{2}[110]$, which is obtained by drawing burger circuit, lies in (001) plane. Since {001}planes are not the gliding planes, Lomer dislocation is hardly mobile. Inverse FFT images of Lomer dislocation are shown in Fig. 2(c2) and (c3), by masking $\{(111)(\bar{1}\bar{1}\bar{1})\}$and $\{(11\bar{1})(\bar{1}\bar{1}1)\}$ planes in FFT pattern, respectively. Two extra half planes were observed at different {111} planes. The Lomer dislocation could be treated as the reaction of two 60° dislocations from different {111} planes: $\,\frac{a}{2}[10\bar{1}]+\,\frac{a}{2}[011]=\,\frac{a}{2}[110]$^30^. It is an energetically favorable process according to Frank’s *b*^2^ criteria: $\frac{{a}^{2}}{2}+\frac{{a}^{2}}{2} > \frac{{a}^{2}}{2}$. Lomer edge dislocation is twice effective for strain relaxation in comparison with that of the 60° mixed type because the efficiency of relaxation scales with the length of edge component of burger vector projected into the interface^29^. However, due to high nucleation energy, the onset of Lomer dislocation is kinetically limited for GeSn epitaxy at low temperature growth.

The zigzagged intrinsic stacking faults were also observed at the GeSn/Ge interface of sample B as shown in Fig. 3(a). The stacking faults start from the GeSn/Ge interface, and then wander through both Ge and GeSn along {111} planes, and end at the GeSn/Ge interface. The stretching angle between two stacking faults is 54.7°. The inset shows the corresponding FFT pattern, in which the streaks along [111] direction indicate the formation of stacking faults. In order to investigate the formation mechanism of stacking faults, two areas were specifically studied, as annotated as area A and area B in Fig. 3(a). Magnified image of area A shown in Fig. 3(b) indicates the presence of Frank partial dislocation at the interface, verified by drawing burger circuit. The stacking fault associated with Frank partial dislocation was also observed at GeSn alloy in the inserted inverse FFT images of Fig. 3(b), which was obtained by masking $\{(11\bar{1})(\bar{1}\bar{1}1)\}$ planes in FFT pattern. As stacking fault extends into GeSn, a 90°Shockley partial dislocation with burger vector$\,\frac{a\,}{6}[11\bar{2}]$ is formed at the edge of stacking fault. Shockley partial dislocation glides to the interface and reacts with Frank partial dislocation to form a perfect Lomer dislocation. Therefore, stacking fault is terminated after this reaction^31^. The reaction is given as:

1 $$\frac{a}{6}[11\bar{2}]({\rm{Shockley}})+\frac{a}{3}[111]({\rm{Frank}})\to \frac{a}{2}[110]({\rm{Lomer}})$$

On the Thompsons tetrahedron diagram, shown as Fig. 3(c), it is marked as,

2 $$D\delta +\delta C\to DC$$

It should be noted that during the reaction no energy reduction occurs before and after this reaction according to Frank’s criteria:$\,\frac{{a}^{2}}{6}+\frac{{a}^{2}}{3}=\frac{{a}^{2}}{2}$. However, there is a significant energy reduction when stacking fault is terminated during the reaction. Therefore, the overall energy of Frank, Shockley partials and stacking faults is greater than the energy of perfect Lomer dislocation, indicating that this process is energetically favorable.

Another mechanism of stacking fault associated with 60° dislocation dissociation was studied as well. The magnified image of area B shown in Fig. 3(d) presents the stacking fault extending into Ge buffer along (111) plane with the length of 9 nm. Using burger circuit enclosing the stacking faults, the projected burger vector $\frac{a\,}{4}[112]$ is determined on the $(\bar{1}10)$ plane, referring to 60° mixed dislocation. It indicates that the 60° dislocation dissociates into a pair of glissile 30° and 90° Shockley partial dislocations bound by stacking fault^32^. The reaction could be written as,

3 $$\frac{a}{2}[101](60^\circ )\to \frac{a}{6}[2\bar{1}1](30^\circ )+\frac{a}{6}[112](90^\circ )$$

or

4 $$BC(60^\circ )\to B\delta (30^\circ )+\delta C(90^\circ )$$

on the Thompsons tetrahedron diagram of Fig. 3(c).

The total energy after the dissociation is one third lower than that of 60° dislocation, comparing the energy states of pre- and post- reaction. For the dissociation of 60° dislocation, the glide motion of top atoms in (111) plane was illustrated in Fig. 3(e) with burger vector $\overrightarrow{b}$ marked. Since the glide of 60° dislocation experiences higher energy barrier than 30° or 90° partial dislocations, it will decompose into the glide of 30° and 90° partial dislocation with the following sequence. The 30° partial dislocation glide takes the lead and 90° partial dislocation closely follows in order to maintain the close packing structure^33^.

### Self-organized dislocation network

**Figure 4:**
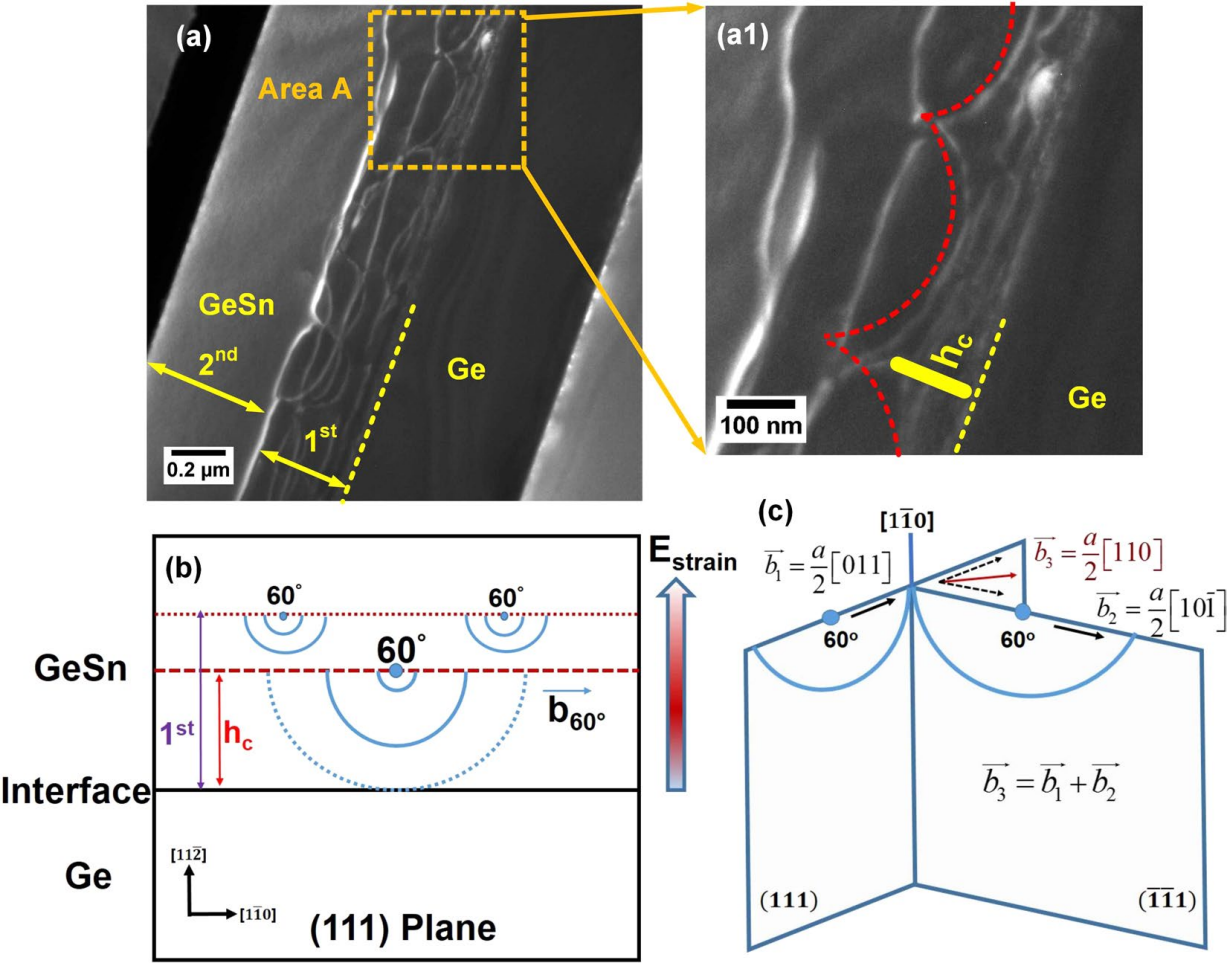
(**a**) Dark field TEM images of sample B indicates the formation of self-organized dislocation network. (**a1**) Zoom-in TEM image of area A exhibits the nucleation of half loops. (**b**) The schematic diagram illustrates half loop nucleation and propagation of 60° dislocation at critical thickness along (111) plane. (**c**) Lomer dislocation is formed with the following reaction: $\frac{a}{2}[011]+\frac{a}{2}[10\bar{1}]=\,\frac{a}{2}[110]$ when two 60° dislocations on $(111)$ and $(\bar{1}\bar{1}1)$ planes intersect with each other.

After the initial nucleation of dislocations at GeSn/Ge interface, the subsequent growth is pseudomorphic within critical thickness. Meanwhile, elastic strain energy accumulates with increasing thickness which impedes the Sn incorporation. Beyond the critical thickness, pseudomorphic epitaxy collapses and dislocations are generated to release the strain energy. As a result, more Sn atoms are incorporated into lattice sites. After the generation of dislocations, a self-organized dislocation network is formed as the result of dislocation propagations and reactions. The typical dark field TEM image of sample B, as shown in Fig. 4(a), confirms the formation of self-organized dislocation network, which was marked as the 1^st^ layer. The 2^nd^ layer is low-defect, suggesting that the majority of dislocations are localized in the 1^st^ layer.

The formation mechanism of dislocation network could be explained by the following process: (i) half-loop nucleation of 60° dislocation, (ii) half loop propagation and (iii) formation of Lomer dislocation. Magnified TEM image of area A shown in Fig. 4(a1) exhibits the nucleation of half loops, which is attributed to the generation of 60° dislocation^34^. The schematic diagram of half-loop nucleation of 60° dislocation was drawn in Fig. 4(b). In the diagram, the 60° mixed dislocations are dominantly nucleated on the epitaxial surface at critical thickness due to its low activation energy. Afterwards, the 60° dislocations will elongate along {111} planes as semicircular half loops^34,35^. Normally the half loops increase the radius continuously due to the strain field, which would eventually reach the GeSn/Ge interface and then form the linear misfit segments at the interface and two arms travelling upwards as threading dislocations^34^. However, another mechanism occurs before the half loops arrive at the interface: When two 60° dislocations glide along different {111}planes and meet with each other, they will intersect by cross-slipping mechanism in which one of the 60° dislocations climbs to another plane^35^. If two 60° dislocations have appropriate burger vectors, they will react and form Lomer dislocation. The threading components of two 60° mixed dislocations annihilate after the reaction without having to travel through the film. This process facilities strain relaxation since Lomer dislocation is more efficient of relieving strain energy.

The total eight equivalent reactions on the pair of {111} planes have been studied and summarized in ref.^36^. One typical reaction was schematically drawn in Fig. 4(c). One 60° dislocation on the (111)planes with ${\overrightarrow{b}}_{1}=\frac{a}{2}[011]$ reacts with another 60° dislocation on $(\bar{1}\bar{1}1)$ plane with${\overrightarrow{b}}_{2}=\frac{a}{2}[10\bar{1}]$, resulting in the formation of Lomer dislocation on (001) plane with ${\overrightarrow{b}}_{3}=\frac{a}{2}[110]$. It could be expressed as,

5 $$\frac{a}{2}[011]+\frac{a}{2}[10\bar{1}]=\frac{a}{2}[110]$$

Similar process will repeat with the continuous growth to gradually relieve the residual strain energy. After the formation of Lomer dislocation, a self-assembled dislocation network is formed within the 1^st^ GeSn layer, efficiently accommodating the lattice mismatch between GeSn and Ge. Therefore, the 1^st^ GeSn layer could act as a sacrificial layer with large amount of dislocations, leaving low-defect GeSn in the 2^nd^ layer.

With gradual strain relaxation beyond critical thickness, the Sn gradient GeSn layer is formed spontaneously in the 1^st^ layer, as indicated as region II in Fig. 1. The spontaneous Sn gradient GeSn is crucial to obtain high quality GeSn because of the Hagen-Strunk multiplication mechanism in compositional gradient layer^37,38^. The Hagen-Strunk multiplication will help generate of 60° dislocations with complementary burger vectors^39,40^, which react and form Lomer dislocations as shown in Fig. 4. As a result, threading branches of two adjacent 60° dislocations will cancel out with each other. The ideal arrangement of 60° dislocation helps terminate threading dislocation which would otherwise propagate through the film and deteriorate material quality. It also results in the minimum number of dislocations that are required to relieve strain since the Lomer dislocations are formed during this process, which have more energy-relieving efficiency. Through Hagen-Strunk multiplication the self-organized dislocation network acts as a “filter” of threading dislocations, enabling successive low defect GeSn growth. This process is unique for GeSn compared with other group IV alloys such as SiGe because of the Hagen-Strunk multiplication induced by the formation of spontaneous Sn gradient layer.

## Discussion

In order to in-depth investigate the strain effect on Sn incorporation, especially during the 1^st^ GeSn layer growth, the Gibbs free energy was calculated in both completely relaxed and compressively strained systems for comparison. Gibbs free energy was proven to provide good approximation of thermal stability of alloys under equilibrium condition. The minimization of free energy is the thermodynamic driving force of the stable crystallization. The calculation of Gibbs free energy has been used to study the thermal stability of GeSn and SiGeSn alloys^25,41^. In this work, the elastic strain energy was introduced into Gibbs free energy to estimate thermodynamic properties of compressively strained GeSn alloy. It is revealed that although metastable GeSn alloy was grown under nonequilibrium condition, our calculation of Gibbs free energy provides good description of strain effects on Sn incorporation with good agreement of experimental results.

The calculation assumes: (i) Thermodynamic equilibrium condition, (ii) random distribution of Sn atoms in Ge crystal and vibration of lattice constants due to fluctuation of alloy mixing negligible, (iii) thin film thickness below the critical thickness limit and (iv) Sn oversaturation condition.

The Gibbs free energy could be expressed as

6 $${\rm{\Delta }}G(x,T)={\rm{\Delta }}H(x,T)-T{\rm{\Delta }}S(x,T)+{E}_{s}(x)$$

where *x* and *T* are Sn composition and system temperature, respectively, Δ*H* and Δ*S* are mixing enthalpy and entropy while *E*_*s*_ is elastic strain energy per atom.

The ideal enthalpy and entropy are given as

7 $${\rm{\Delta }}H(x,T)=\alpha x(1-x)$$

8 $$T{\rm{\Delta }}S(x,T)=-kT[xIn(x)+(1-x)In(1-x)]$$

where *α* is interaction parameter which scales proportionally with the square of bond length between two nearest-neighbor atoms^42^. The value of *α* could be obtained experimentally by fitting of liquidus and curves in the $T-x$ phase diagram of the alloy system (Supplementary section 5).

The elastic strain energy per atom is written as^43^

9 $${E}_{s}(x)=2\mu \frac{1+v}{1-v}{f}^{2}\,$$

Hereby, *μ* is shear modulus and *v* is Poisson’s ratio. Within critical thickness, the value of strain *f* could be simplified as$\frac{{a}_{GeSn}-{a}_{Ge}}{{a}_{GeSn}}$, where ${a}_{GeSn},\,{a}_{Ge}$ are lattice constants of GeSn and Ge, respectively. Detailed calculations of the whole range *f* were provided in supplementary section 6. The relevant parameters for GeSn system were listed in Supplementary Table S1 of section 7.

**Figure 5:**
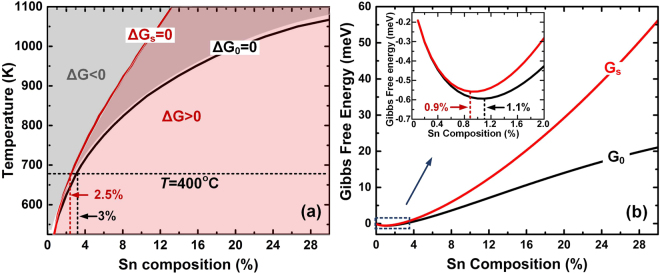
(**a**) Plots of Gibbs free energy *ΔG*_0_ = 0 and *ΔG*_s_ = 0 correspond to the stability boundary of fully relaxed and compressively strained GeSn system, respectively. The grey and purple zone marked in the plots represent the stable (Δ*G* > 0) and unstable (Δ*G* > 0) region. Under compressive strain, stability boundary (*ΔG*_*s*_ = 0) shifts to lower Sn composition, shrinking the stable region. At *T* = 400 °C, the maximum Sn composition for strained and unstrained system is 2.5% and 3%, respectively. (**b**) Gibbs free energy plot at *T* = 400 °C with (*G*_*s*_) and without strain (*G*_0_). The local minimum Sn contents for strained system and unstrained system occur at 0.9% and 1.1%, respectively.

The Gibbs free energy ${\Delta }{G}_{0}=0\,{\rm{and}}\,{\Delta }{G}_{s}=0\,$ are shown in phase diagram of Fig. 5(a), representing the stability boundary of unstrained (fully relaxed) and compressively strained GeSn alloy systems, respectively. Hereby, the stability area (*ΔG* < 0) was marked as grey field while instability area (Δ*G* > 0) was colored as purple field. Compared with unstrained curve Δ*G*_0_ = 0, the whole Δ*G*_*s*_ = 0 curve shifts to the lower Sn composition range because of elastic strain energy, thus narrowing the stability area. As a result, the maximum Sn incorporation obtained in stable strained GeSn system decreases compared with unstrained system under the same temperature. For *T* = 400 °C, which is the upper limit temperature of our growth, the maximum Sn composition for strained system is 2.5%, smaller than 3% of unstrained one. More discrepancy of maximum Sn composition appears at higher temperature range. Similar composition-stability relationship was further clarified by the calculation of Gibbs free energy *G*(*x*,*T*) at *T* = 400 °C, which was plotted in Fig. 5(b). As shown in the inserted zoomed-in curves, the minimum free energy for strained and unstrained system occurs at 0.9% and 1.1% Sn composition, respectively. Since Δ*G* < 0 is the driving force of stable crystallization, Sn atoms tend to incorporate less in strained GeSn system compared to unstrained one in order to minimize Gibbs free energy. The more strained energy accumulates, the less Sn incorporates into GeSn system.

In the 2^nd^ layer of GeSn, the relationship between compressive strain and Sn incorporation are interactive. As discussed above, more Sn could be incorporated with gradual relaxation of compressive strain, leading to the increase of local microscopic lattice constant. Therefore, more compressive strain is introduced for successive epitaxy of several atomic layers, which in turn impedes Sn incorporation. The interplay between strain and Sn incorporation delays the occurrence of maximum Sn incorporation and results in the long Sn-enhanced tail with continuous growth. Although the world-record maximum Sn composition of 22.3 % has been achieved in this study, the steep gradient rates at 2^nd^ layer shown in SMIS of Fig. 1 suggest that Sn incorporation is far from saturation at the end of GeSn growth. The further continuous growth with the same recipe would lead to the “true” maximum Sn composition which is eventually limited by surface chemical reaction. The detailed analysis is under investigation and will be reported later.

## Conclusion

Two growth strategies were investigated in order to analyze strain relaxation, high Sn incorporation, and their relationship. Compressive strain has a strong effect on Sn incorporation, which is well explained by Gibbs free energy calculation including elastic strain energy. The gradual strain relaxation results in spontaneous formation of gradient GeSn. Step graded GeSn structure relaxes compressive strain more smoothly and the final Sn incorporation of 22.3% was achieved, which is unprecedented so far for CVD technology. Different dislocation arrangements are revealed at GeSn/Ge interface. The mixed type 60° dislocations are dominant at the interface over Lomer dislocations. Intrinsic stacking faults are associated with two different reactions: 60° dislocation dissociation and Lomer dislocation formation. Beyond critical thickness half loops of 60° dislocations are nucleated and expended outwards as threading dislocations. Spontaneous gradient GeSn helps terminating threading dislocation by Hagen-Strunk multiplication. The well-ordered dislocation network was formed at gradient layer, leading to the low-defect GeSn on the top. This work provides the thorough analysis of strain relaxation mechanism of GeSn and offers an essential guidance for low defect GeSn growth with high Sn content.

## Methods

### CVD growth

Ge buffer layer with approximately 600 nm thickness was grown by low/high temperature two-step growth followed by post thermal annealing. The 1^st^ 150 nm-thick layer was grown at temperature of 375 °C while the 2^nd^ 450 nm-thick layer was grown at temperature of 600  °C using 10% GeH_4_ in purified H_2_ carrier gas. Afterwards the *in-situ* annealing was done at >800  °C. GeSn growth was initiated on Ge buffer with the temperature between 200 and 400 °C. SnCl_4_ is a liquid at room temperature and must be delivered using a bubbler in which H_2_ gas is metered to control the SnCl_4_ mass flow rate. All the sample was grown with a SnCl_4_/(GeH_4_+SnCl_4_+H_2_) molar flow fraction of 10^−5^ order of magnitude. For sample E three-step Sn-enhanced layer structure was grown with target thickness 100 nm for each step layer. The SnCl_4_ flow fraction for each step epitaxy increases by ~8% compared with the precious step.

### TEM imaging

The specimen of TEM was mechanically polished until thickness was down to 20 μm. It was then transferred onto a copper grid with 2 mm-diameter concentric hole. Focused ion beam thinning was followed by Fischione 1010 low-angle ion milling machine. The final thickness of TEM-observing area is 50–300 nm. Cross-sectional TEM images were observed using a Cs corrected Titan 80–300 with a Schottky field emission gun (FEG) operating at 300 kV.

## Electronic supplementary material

Supplementary Information
