# Supplementary material for: Investigation of GeSn Strain Relaxation and Spontaneous Composition Gradient for Low-Defect and High-Sn Alloy Growth
Source: Sci Rep. 2018 Apr 4;8:5640. doi: 10.1038/s41598-018-24018-6 (PMC5884787; doi:10.1038/s41598-018-24018-6)
Supplement: Supplementary file 1 — Supplementary Information [file 41598_2018_24018_MOESM1_ESM.docx]

**SUPPLEMENTARY INFORMATION**

**Investigation of GeSn Strain Relaxation and Spontaneous Composition Gradient for Low-Defect and High-Sn Alloy Growth**

Wei Dou^1^, Mourad Benamara^2^, Aboozar Mosleh^3,4^, Joe Margetis^5^, Perry Grant^3,6^, Yiyin Zhou^3,6^, Sattar Al-Kabi^3^, Wei Du^7,8^, John Tolle^5^, Baohua Li^6^, Mansour Mortazavi^7^, Shui-Qing Yu^1*^

^1^Department of Electrical Engineering, University of Arkansas, Fayetteville, AR 72701, USA

^2^ Institute for Nanoscience and Engineering, University of Arkansas, Fayetteville, AR, 72701, USA

^3^Microelectronics-Photonics Program, University of Arkansas, Fayetteville, AR 72701, USA

^4^Department of Electrical Engineering, Arkansas Tech University, Russellville, AR 72801, USA

^5^ASM, 3440 East University Drive, Phoenix, AZ 85034, USA

^6^Arktonics, LLC, 1339 South Pinnacle Drive, Fayetteville, AR 72701, USA

^7^Department of Chemistry & Physics, University of Arkansas at Pine Bluff, Pine Bluff, AR 71601, USA

^8^Department of Electrical Engineering, Wilkes University, 84 West South Street, Wilkes-Barre, PA 18766, USA

^*^[syu@uark.edu](mailto:syu@uark.edu)

This file contains:

1. Summary of EDX-STEM results
2. XRD measurement and analysis
3. Calculation of critical thickness
4. PL spectra and analysis
5. Bindoal and spinodal curves on T-x phase diagram
6. Calculation of elastic energy, effective stress, strain, and relaxation
7. Relevant parameters for theoretical calculation
8. References
9. **Summary of EDX-STEM results**


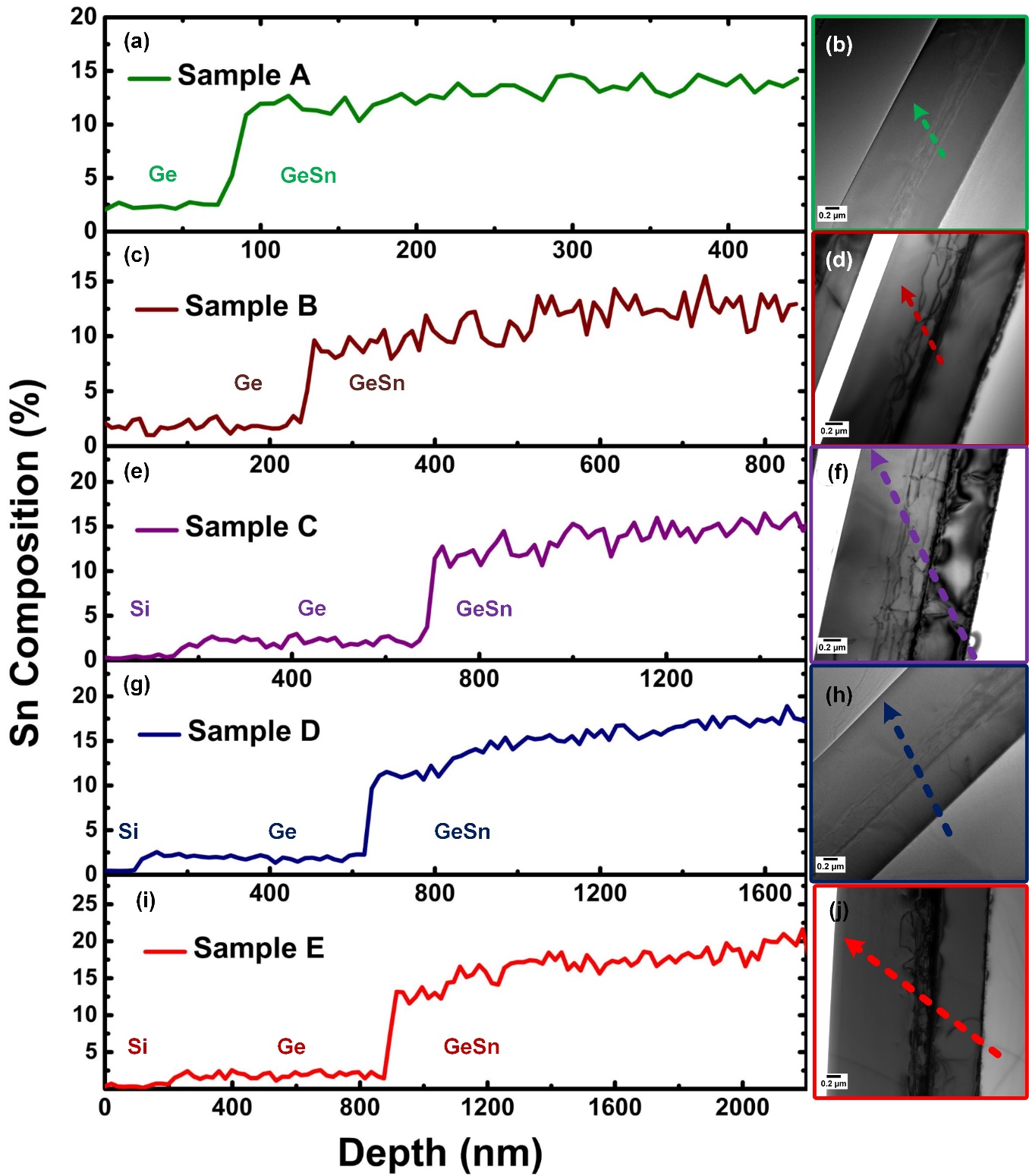


**Figure S1**. EDX-STEM results show Sn composition curves **(a), (c), (e), (g),** and **(i)** as function of scanning depth for samples A, B, C, D, and E, respectively. Bright fields of cross sectional TEM images of sample A, B, C, D, and E viewed from $\left[ \bar{1}10 \right]$ direction are shown in **(b), (d), (f),** **(h)**, and **(j)**, respectively. EDX line scanning paths were also marked in the TEM images. Note that the scanning paths are not parallel to growth direction in order to obtain accurate Sn composition.

1. **XRD measurement and analysis**


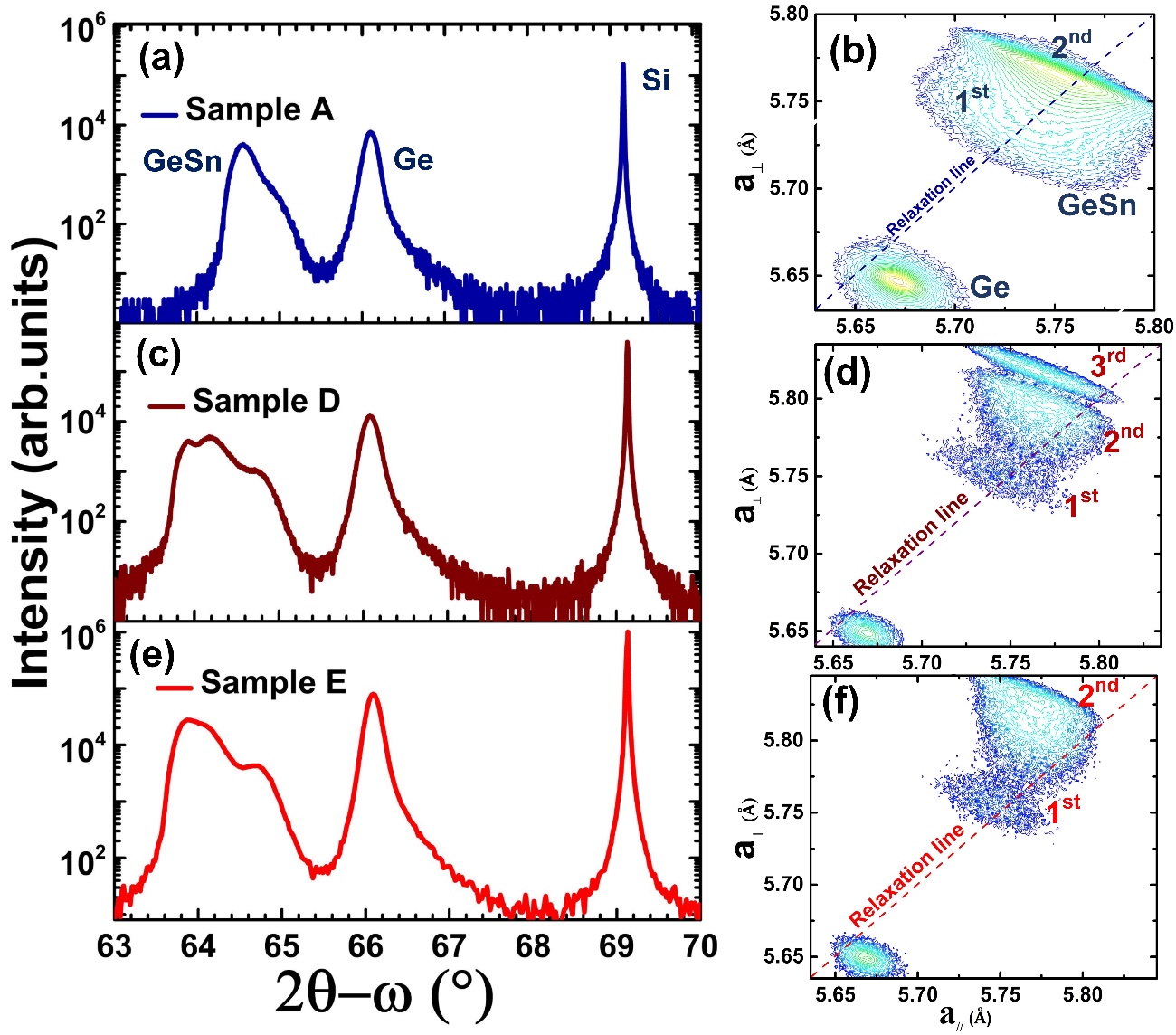


**Figure** **S2**. The symmetric 2ɵ-ω scans **(a)**, **(c),** and **(e)** along (004) plane for sample A, D, and E, respectively. RSMs of XRD **(b)**, **(d),** and **(f)** at the asymmetric (224) for sample A, D, and E, respectively. In RSM, a_//_ and a_┴_ correspond to the in-plane and out-of-plane lattice constants, respectively. Relaxation line and layer information were marked in the RSMs.

The symmetric XRD 2ɵ-ω scans along (004) plane of sample A, D, and E were plotted in Fig. S2 (a), (c), and (e), respectively. The corresponding RSM color contours at the asymmetric (224) scan were drawn in Fig. S2 (b), (d), and (f) for sample A, D, and E, respectively. XRD was performed using a Philips X’pert MRD system, which was equipped with a standard four-bounce Ge (220) monochromator and a three bounce (022) channel cut Ge analyzer crystal along with the 1. 6 kW Cu Kα1 X-ray tube with vertical line focus. Two-layer structure was observed for sample A while three-layer characteristic was shown in sample D. For sample E, the three-step Sn compositional graded structure was not resolved. The layer structure information from XRD shows high consistency with TEM and SIMS results. The least square fitting was performed to determine the average Sn compositions and strains for each layer. Vegard’s law was employed to calculate the relaxed lattice constant of GeSn: $a_{GeSn}=(1-x)a_{Ge}+xa_{Sn}+bx(1-x)$, where $a_{GeSn}$, $a_{Ge}$, and $a_{Sn}$ are lattice constants of GeSn, Ge and *α*-Sn, respectively, $x$ is Sn composition and $b$ is bowing parameter, which has been reported by various literatures^1-4^. In this work, the bowing parameter $b=-0.066 Å$ was adopted^4^, which has been proven to best fit our experimental result. The strain information obtained by fitting was listed in Table 1. It should be noted that the Ge buffer layer undergoes tensile strain up to 0.25%, which is due to the discrepancy of thermal expansion coefficients between Si and Ge. After cooling down from thermal annealing (*T*=800°C), residual tensile strain remains in Ge buffer layer.

1. **Calculation of critical thickness**

The critical thickness *h_c_* of GeSn alloy on Ge was calculated using both Matthews and Blakeslee (M- B) model and People and Bean (P- B) model, as shown in Fig. S3.

The critical thickness based on M- B model is written as^5^,

${h_{c}}^{M-B}=\frac{1}{8\pi}\frac{b}{f}\left[ \frac{1-v{cos}^{2}\beta}{\left( 1+v \right)cos\lambda} \right][\ln\frac{{h_{c}}^{M-B}}{b}+1]$ (1)

where $b$ is the burger vector, $f$ is the strain value, $v$ is poisson’s ratio, $\beta$ is the angle between slip plane and the direction perpendicular to intersection line of interface and slip plane and $\lambda$ is the angle between burger vector $b$ and dislocation line. For GeSn/Ge system, $b$ is the burger vector of 60° mixed dislocation since it dominates in GeSn epitaxy. So$cos\beta= cos\lambda=0.5$. The value of strain $f$ could be estimated as$\frac{a_{GeSn}-a_{Ge}}{a_{GeSn}}$, where $a_{GeSn}, a_{Ge}$ are lattice constants of GeSn and Ge, respectively. Poission ratio $v$ could be obtained by linear interpolation between Ge and *α*-Sn. Matthews and Blakeslee (M- B) model is also called mechanical equilibrium model. It describes that misfit stress drives the preexisting threading dislocations to elongate and form MDs segments^5^. However, this model is not sufficient to describe the epitaxy that has low interfacial MDs.

People and Bean (P- B) model is based on the energy balance of non-equilibrium theory where the areal strain energy density is balanced against self-energy of an isolated dislocation^6^. The film was grown initially free of MDs until the accumulated elastic strain energy exceeds activation energy of MDs. Thereafter, the dislocations nucleate at the critical thickness *h_c_* from the interface.

Based on P- B model, the critical thickness could be revised as^6^

${h_{c}}^{P-B}=\frac{1}{16\sqrt{2}\pi}\frac{b^{2}}{af^{2}}\frac{1-v}{1+v}\ln\frac{{h_{c}}^{P-B}}{b}$ (2)

The experimental critical thickness of GeSn grown on Ge follow the similar trend, but higher than the predication using P- B model^7^, which assumes that the initial grown film is free of MDs. However, based on our analysis of dislocation configuration at GeSn/Ge interface, strain energy is partially relaxed at the initial growth since MDs already generate at the interface. Therefore, the actual critical thickness remains larger than theoretical predication of P- B model.


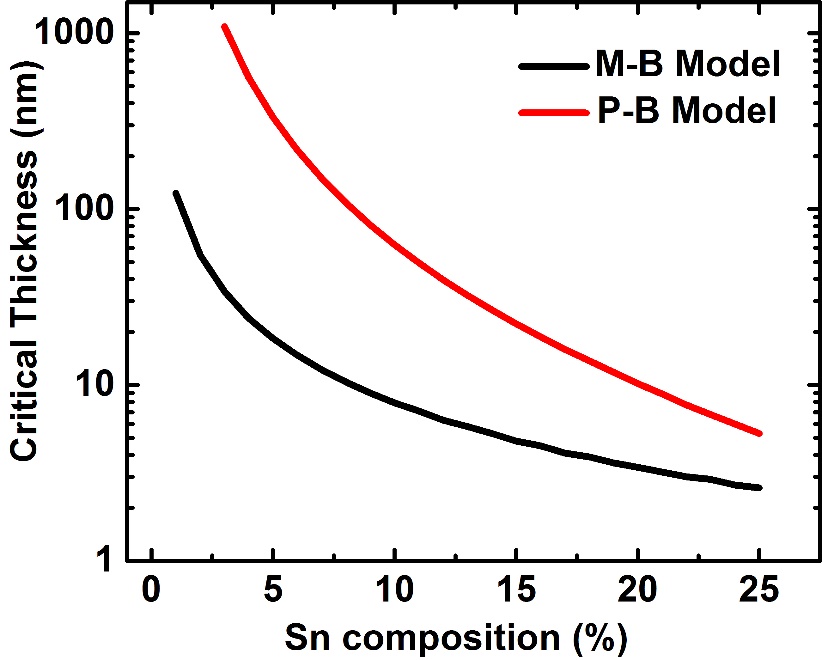


**Figure** **S3**. Critical thicknesses calculation for GeSn on Ge buffer based on Matthews and Blakeslee (M-B) model and People and Bean (P-B) model.

1. **PL spectra and analysis**


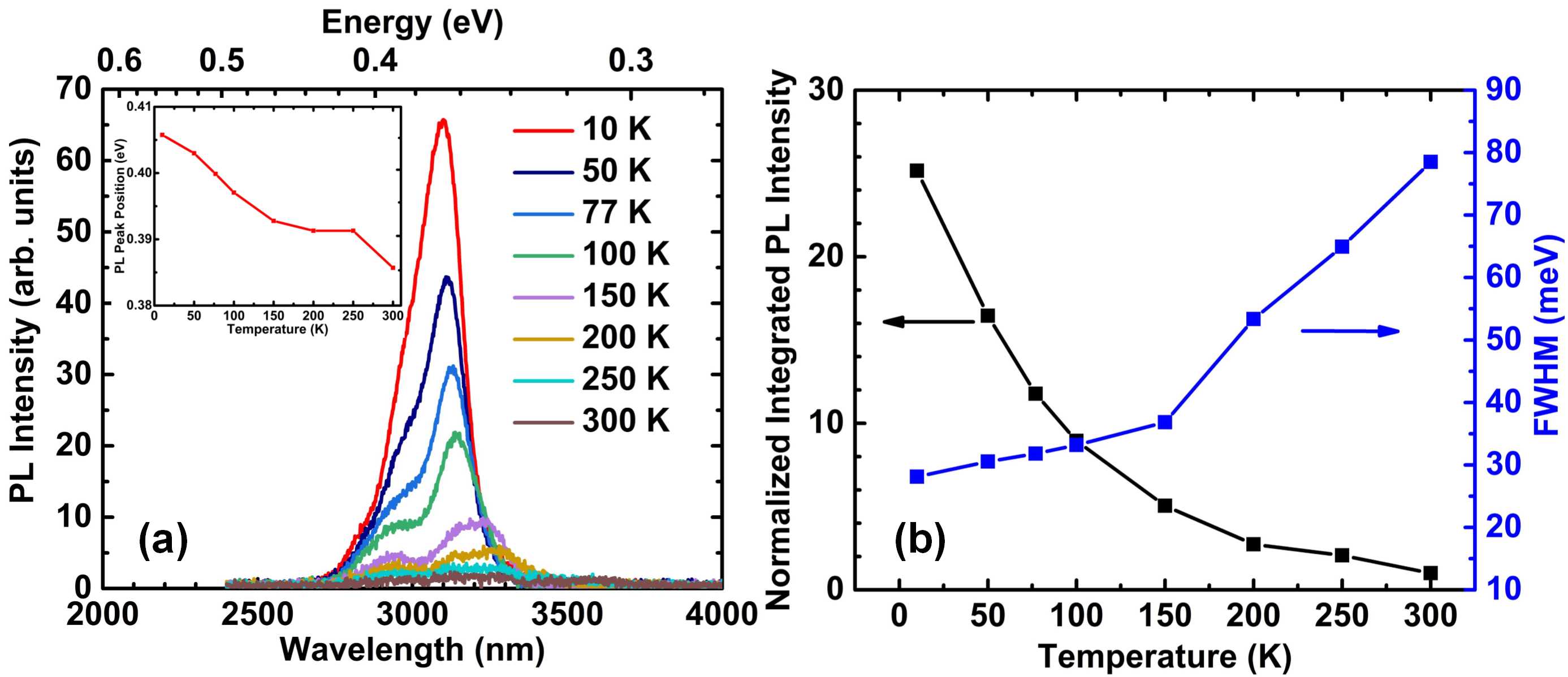


**Figure S4**. **(a)** Temperature dependent photoluminescence of sample E from 10 to 300 K. The insert shows temperature dependent peak shift, following the typical Varshni model. **(b)** The integrated PL intensity increases 25 times with decreasing temperature from 300 to 10 K, featuring the direct bandgap characteristics. The full width of half maximum (FWHM) reduces significantly from 300 to 10 K.

The temperature dependent photoluminescence of sample E was plotted in Fig. S4 (a), with temperature ranging from 10 to 300 K. A continuous wave (CW) laser emitting at 532 nm wavelength was used as optical-pumping source. The pumping power was measured as 50 mW and the beam size was 100 µm in diameter. PL emission was spectrally analyzed by the combination of iHR320 spectroscopy and liquid nitrogen cooled Indium Antimonide (InSb) detector with the wavelength detection cut-off at 5 µm. A SR830 DSP lock-in system along with a chopper was applied to amplify the PL signal. Samples were mounted in a helium-cooled cryostat with temperature ranging from 10 K to 300 K. For sample E, PL spectra exhibit a clear blue shift with decreasing temperature, shown in the insert of Fig. S4 (a). The shift follows classical Varshni empirical model which describes the temperature dependence of bandgap energy of semiconductors. The wavelength is extended up to 3220 nm at 300 K. As shown in Fig. S4 (b), the integrated PL intensity increases 25 times as temperature decreases from 300 to 10 K, featuring the true direct bandgap material characteristics. Meanwhile, the FWHM decreases at lower temperature. The slight absorption from the ambient environment was observed at ~3000 nm wavelength on the spectra.

1. **Bindoal and spinodal curves on** $\boldsymbol{T}\mathbf{-}\boldsymbol{x}$ **phase diagram**


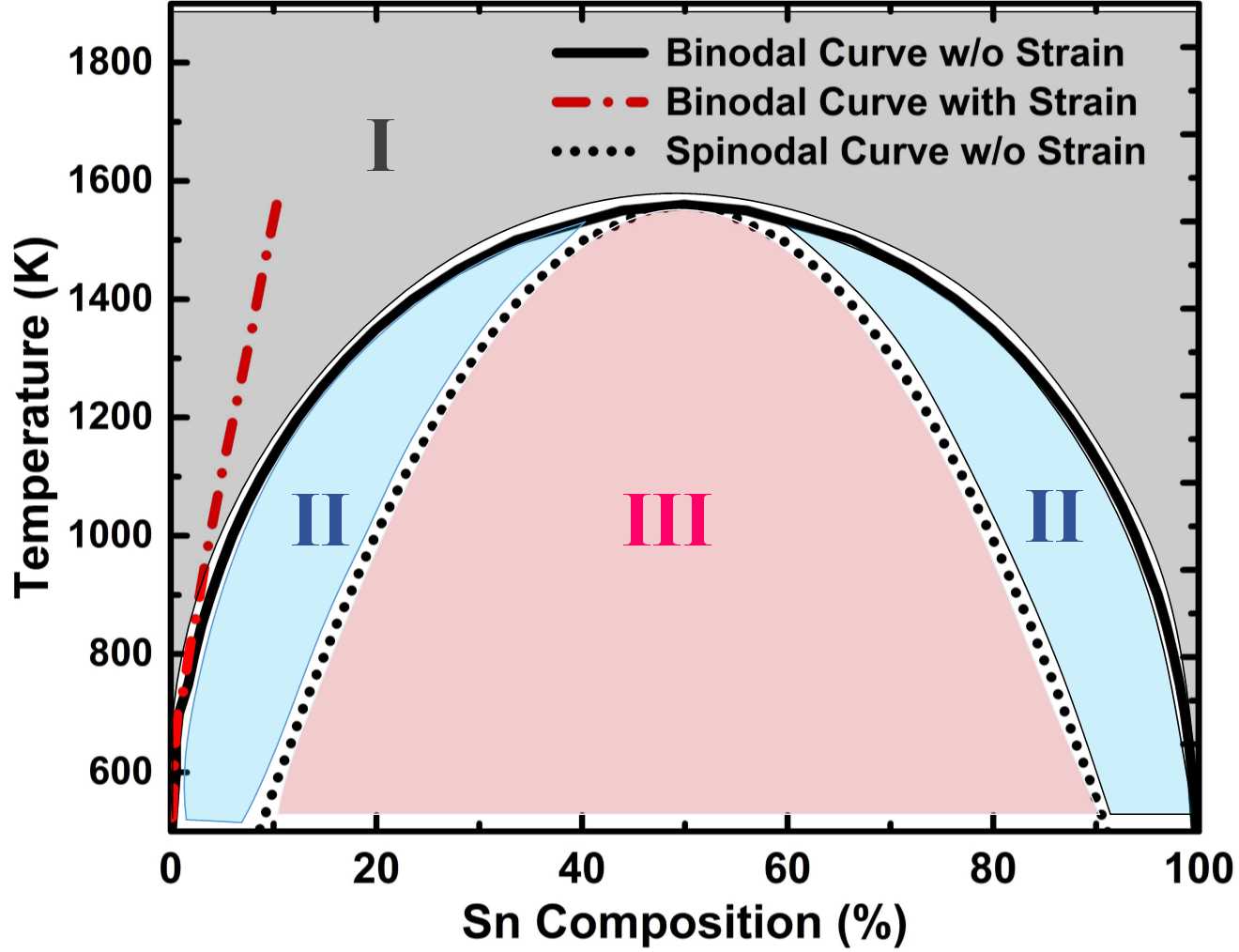


**Figure S5.** Temperature-composition ($T-x$) phase diagram of GeSn was plotted, where the bindoal (black solid) and spinodal (black dotted) curves of unstrained system were calculated using $\frac{\partial G}{\partial x}=0$ and$\frac{\partial^{2}G}{\partial x^{2}}=0$, respectively. *G* is the Gibbs free energy of GeSn system. The bindoal and spinodal curves divide the whole area as three regions: I) stable region, II) metastable region and III) unstable region. The binodal curve of strained GeSn system (red dash dot) was also drawn in the diagram.

The $T-x$ phase diagram was shown in Fig. S5, in which bindoal and spinodal curves for strained system were calculated under equilibrium condition. Bindoal curve represents the equilibrium solubility of Sn at the given temperature, corresponding to Sn composition at the minimum point of Gibbs free energy where$\frac{\partial G}{\partial x}=0$. The spinodal composition was calculated as $\frac{\partial^{2}G}{\partial x^{2}}=0$. Beyond the spinodal composition the system becomes unstable and spinodal decomposition occurs. The critical temperature *T_c_*=1560 K is achieved at the top merging point of both bindoal and spinodal curves, above which the alloy becomes miscible for all the compositions with random Sn distribution. Below critical temperature, the miscibility gap occurs. The $T-x$ diagram is divided into three regions by biondal and spinodal curves: I) Stable region in which GeSn system keeps stable. II) Mestable region. Spontaneous decomposition of GeSn is hampered by the activation energy barrier. III) Unstable region in which GeSn decomposes spinodally. The binodal curve of strained GeSn system is also drawn in the diagram. Comparing to unstrained system, the strained binodal curve shifts to lower Sn composition at the given temperature, which is consistent with the results shown in Fig. 5.

1. **Calculation of elastic energy, effective stress, strain, and relaxation**


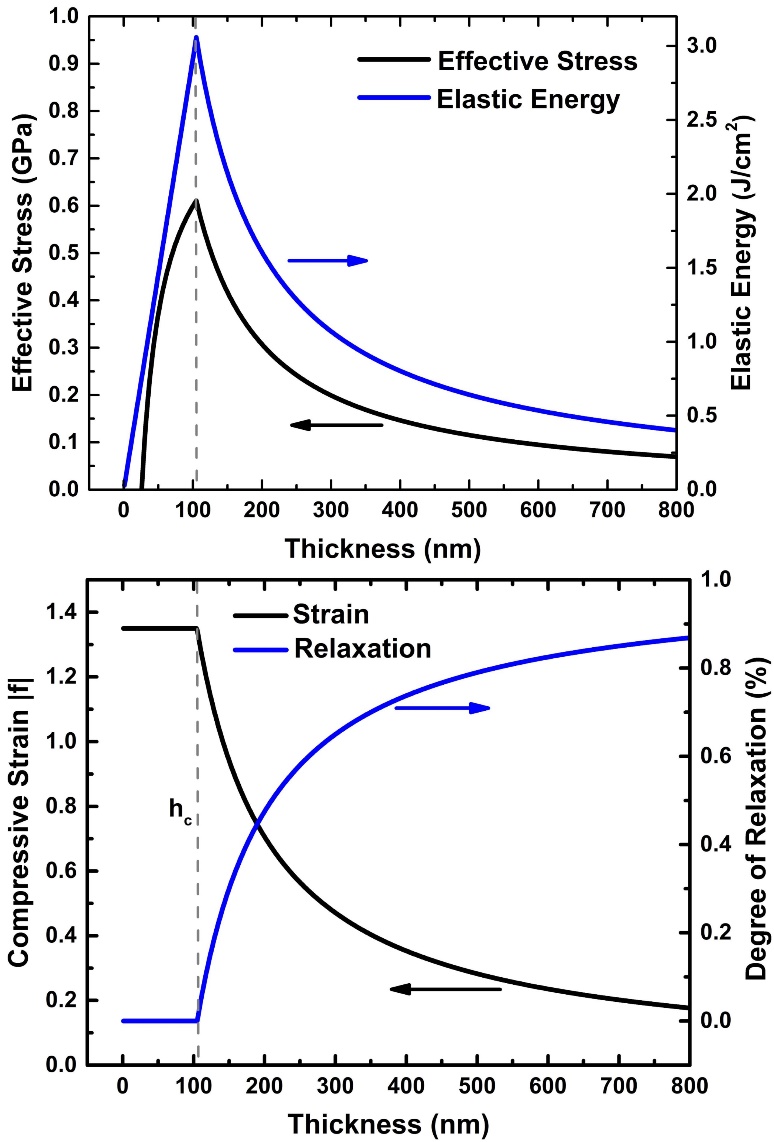


**Figure S6.** **(a)** Elastic energy and effective stress of Sample A were plotted as a function of thickness. **(b)** Strain and relaxation as a function of thickness were drawn. Critical thickness *h_c_* was marked in the curves.

The strain in the film could be expressed as^8^

$f_{1}=\frac{a_{GeSn}-a_{Ge}}{a_{GeSn}} \left( h\leq h_{c} \right)$ (3)

$f_{2}=\frac{1}{8\pi}\frac{(1-v{cos}^{2}\lambda)b^{2}}{(1+v)b_{\|}}\frac{1}{h} \ln(\frac{4R}{b})=\frac{A}{h} \left( h>h_{c} \right)$ (4)

where $R$ is the external cut off radius of dislocation loop, $b_{\|}$is the edge component of burger vector parallel to the interface. Using boundary condition at critical thickness *h_c_*, the constant of *A* could be determined as:$A=f_{1}*h_{c}$. The parameters used in the calculation were given in Table S1, using linear interpolation method. Fig. S2 (a) shows the effective stress and elastic energy of sample A as a function of thickness. Below critical thickness, effective stress linearly increases with thickness. Meanwhile, elastic strain energy accumulates. Beyond the critical thickness both effective stress and elastic energy drop rapidly, inversely proportional to the thickness because of the formation of large amounts of MDs. Both strain energy and effective stress reach the maximum at the point of critical thickness *h_c_*.

The expression of degree of relaxation is $R=\frac{a_{\|}-a_{Ge}}{a_{GeSn-a_{Ge}}}$. $R$could also be expressed by strain$f$, which is written as

$R=\frac{a_{GeSn}(1-|f|)-a_{Ge}}{a_{GeSn}-a_{Ge}}$ (5)

The effective shear stress is given as^9^

$\tau_{eff}=ucos\beta\frac{1-v}{1+v}f-\frac{ucos\beta}{8\pi}bcos\lambda\frac{1-v{cos}^{2}\beta}{1-v}\ln\frac{4h}{b}$ (6)

The elastic strain energy is calculated as^10^

$E_{elastic}=2u\frac{1+v}{1-v}f^{2}t$ (7)

where $t$ is the film thickness. As shown in Fig. S2 (b), below critical thickness the strain remains constant and beyond that strain is reversely proportional to the thickness because of the generation of dislocation network. The value of strain becomes steady gradually with continuous growth.

1. **Relevant parameters for theoretical calculation**

**Table** S1. Summary of Relevant parameters for GeSn alloys.

|  | Ge | Sn |
| --- | --- | --- |
| Lattice constant *a* (nm) | 0.56573^11^ | 0.64892^11^ |
| Bowing parameter *b* (nm) | -0.0066^1^ | |
| Elastic constants *C_11_* (GPa) | 128.53^11^ | 69.00^11^ |
| Elastic constants *C_12_* (GPa) | 48.26^11^ | 29.30^11^ |
| Elastic constants *C_44_* (GPa) | 68.30^11^ | 36.20^11^ |
| Calculated Poisson ratio  $v=\frac{C_{12}}{C_{11}+C_{12}}$ | 0.27 | 0.30 |
| Shear modulus *µ* (GPa)^12^  $\mu=C_{44}-\frac{(2C_{44}+C_{12}-C_{11})}{3}$ | 49.52 | 25.30 |
| Interaction parameter *α* (eV) | 0.27^13,14^ | |

1. **References**
2. Beeler, R., Roucka, R., Chizmeshya, A., Kouvetakis, J. & Menéndez, J. Nonlinear structure-composition relationships in the Ge_1− y_Sn_y_/Si (100) (y<0.15) system. *Phys. Rev. B* **84**, 035204 (2011).
3. Gencarelli, F. *et al.* Crystalline properties and strain relaxation mechanism of CVD grown GeSn. *ECS J. Solid State Sci. Technol.* **2**, P134-P137 (2013).
4. Kouvetakis, J., Menendez, J. & Chizmeshya, A. Tin-based group IV semiconductors: New platforms for opto-and microelectronics on silicon. *Annu. Rev. Mater. Res.* **36**, 497-554 (2006).
5. Chibane, Y. & Ferhat, M. Electronic structure of Sn_x_Ge_1−x_ alloys for small Sn compositions: unusual structural and electronic properties. *J. Appl. Phys.* **107**, 053512 (2010).
6. Matthews, J. & Blakeslee, A. Defects in epitaxial multilayers: I. MDs. *J. Cryst. Growth* **27**, 118-125 (1974).
7. People, R. & Bean, J. Calculation of critical layer thickness versus lattice mismatch for Ge_x_Si_1−x_/Si strained‐layer heterostructures. *Appl. Phys. Lett.* **47**, 322-324 (1985).
8. Wang, W., Zhou, Q., Dong, Y., Tok, E. & Yeo, Y. Critical thickness for strain relaxation of Ge_1−x_Sn_x_ (x ≤ 0.17) grown by molecular beam epitaxy on Ge (001). *Appl. Phys. Lett.* **106**, 232106 (2015).
9. Beanland, R., Dunstan, D. & Goodhew, P. Plastic relaxation and relaxed buffer layers for semiconductor epitaxy. *Adv. Phys.* **45**, 87-146 (1996).
10. Houghton, D. Strain relaxation kinetics in Si_1−x_ Ge_x_/Si heterostructures. *J. Appl. Phys.* **70**, 2136-2151 (1991).
11. Nabarro, F. R. Theory of crystal dislocations. (1967).
12. Madelung, O. Semiconductors: Group IV elements and III-V compounds. (*Springer Science & Business Media*, 2012).
13. Fitzgerald, E. GeSi/Si Nanostructures. *Annu. Rev. Mater. Sci.* **25**, 417-454 (1995).
14. Martins, J. L. & Zunger, A. Bond lengths around isovalent impurities and in semiconductor solid solutions. *Phys. Rev. B* **30**, 6217 (1984).
15. Xie, J. *et al.* Synthesis, Stability Range, and Fundamental Properties of Si−Ge−Sn Semiconductors Grown Directly on Si (100) and Ge (100) Platforms. *Chem. Mater.* **22**, 3779-3789 (2010).
